# Relationship between expression and prognostic ability of PTEN, STAT3 and VEGF-C in colorectal cancer

**DOI:** 10.3892/etm.2012.651

**Published:** 2012-07-31

**Authors:** CANHUI JIN, AIHONG WANG, JIANMIN CHEN, XIAOMIN LIU, GONGPING WANG

**Affiliations:** The First Affiliated Hospital of Henan University of Science and Technology, Luoyang, Henan 471003, P.R. China

**Keywords:** colorectal cancer, prognosis, PTEN, STAT3, VEGF-C, immunohistochemistry

## Abstract

Expression of phosphatase and tensin homolog deleted on chromosome 10 (PTEN), signal transducer and activator of transcription-3 (STAT3) and vascular endothelial growth factor-C (VEGF-C) and their relationship with clinico-pathological features and prognostic ability was determined using immunohistochemistry in 68 cases of colorectal cancer with follow-up data. Kaplan-Meier survival analysis was performed and the prognostic value was determined using univariate analysis. PTEN, STAT3 and VEGF-C expression was detected in 32.4, 60.3 and 63.2% of colorectal carcinoma cases and 90.0, 0 and 0% of normal colon samples, respectively. PTEN and STAT3 were correlated with pathological grade (p=0.011, p=0.001, respectively), but not with tumor size, lymph node metastasis or clinical stage. VEGF-C was correlated with lymph node metastasis (p=0.002), but not with tumor size, pathological grade or clinical stage. Expression of STAT3 and VEGF-C was negatively correlated with PTEN (r=−0.402, r=−0.320, respectively), whereas STAT3 and VEGF-C expression was positively correlated with PTEN (r=0.254). The 3- and 5-year survival rates of PTEN protein-positive patients (68.1 and 50.0%, respectively) were significantly higher than those of PTEN protein-negative patients (32.6 and 19.6%, respectively; p=0.008). The 3- and 5-year survival rates of STAT3-positive (29.3 and 17.1%, respectively) were significantly lower than those of STAT3-negative patients (66.7 and 48.1%, respectively; p=0.005). The 3- and 5-year survival rates of VEGF-C-positive patients (29.3 and 17.1%, respectively) were significantly lower than the rates of VEGF-C-negative patients (66.7 and 48.1%, respectively; p=0.003, p=0.004, respectively). Multivariate analysis revealed that VEGF-C expression was an independent prognostic factor. In conclusion, this study indicates that PTEN, STAT3 and VEGF-C expression are beneficial prognostic factors, which may aid in the accurate assessment of prognosis and guide clinical treatment of colorectal cancer patients.

## Introduction

Colorectal cancer is the world’s third most common malignant tumor, after lung and breast cancer and is associated with a poor prognosis (1). The incidence of colorectal cancer is rising (2) and patients often present in the later stages. Tumor invasion and metastasis are the leading cause of death in colorectal cancer, therefore identification of those factors that regulate colorectal cancer metastasis, prognosis and optimal treatment are of great significance. The activation of oncogenes and the inactivation or loss of function of tumor-suppressor genes are important steps in colorectal carcinogenesis; however, to date, no sensitive and specific biological indicators of malignancy and prognosis exist for colorectal cancer.

Tumor development, invasion and metastasis are dependent on signaling between cells and lymphangiogenesis. In recent years, the effect of deletion of the chromosome 10 phosphatase and tensin homolog gene (PTEN), and expression of signal transducer and activator of transcription-3 (STAT3) and vascular endothelial growth factor-C (VEGF-C) have been investigated in malignant tumors (3,4). PTEN exerts protein phosphatase activity to inhibit Ras-mediated MAPK signaling cascades, thereby reducing STAT3 transcriptional activity and inhibiting cell migration and invasion (3). Klatte *et al* proposed that PTEN inhibits the expression of VEGF-C in renal cell carcinoma (4), while Lee *et al* demonstrated that VEGF-C is a downstream target gene under the regulation of STAT3 (5).

Despite these advances, the role of PTEN, STAT3 and VEGF-C in colorectal cancer has yet to be reported. In the present study, we examined PTEN, STAT3 and VEGF-C protein expression in colorectal cancer and analyzed their prognostic ability.

## Materials and methods

### Colorectal tumor samples

We selected tissue samples from 68 cases of colorectal adenocarcinoma undergoing radical surgery at the First Affiliated Hospital of Henan University of Science and Technology between June 2004 and October 2005. Written informed consent was obtained from each patient and Ethics Committee approval from our institution was obtained. All specimens were confirmed by pathology, and complete clinical data and follow-up records were available to the follow-up deadline of December 2010. For all patients, surgery was the first treatment for cancer. In total, there were 40 males and 28 females, aged between 32 and 78 years; median age was 57 years. A total of 39 patients received a FOLFOX4 chemotherapy scheme after surgery and completed the chemotherapy, 12 patients did not have chemotherapy and 17 only took it irregularly. Normal control colorectal tissues were obtained from 20 individuals during surgery for other diseases.

### Reagents

The Universal SP immunohistochemistry kit, DAB kit color, mouse anti-human PTEN monoclonal antibodies, rabbit anti-STAT3 monoclonal antibodies and rabbit anti-human VEGF-C polyclonal antibody were purchased from Sigma, St. Louis, MO, USA.

### Immunohistochemistry

Immunohistochemistry was performed using the Universal SP immunohistochemistry kit following the manufacturer’s instructions, followed by moderate hematoxylin staining, dehydration in a graded ethanol series and xylene, after which sections were mounted with neutral gum and observed by light microscopy. The negative control was performed with PBS instead of the primary antibody. The number of positive cells and color intensity was scored using a semi-quantitative method from 5 high-power fields of view (6). Clear brown particles appearing in the cytoplasm or nucleus were recorded as positive cells, and the percentage of positive cells was scored as follows: 0, <10% of cells; 1, 10–49% of cells; 2, 50–74% of cells; 3, >75% of cells. Staining intensity was scored as follows: 0, colorless; 1, light yellow; 2, brown; 3, tan. The scores for the number of stained cells and staining intensity were added together: Scores of 0–2 were considered negative and scores >2 were considered positive.

### Prognostic analysis

Follow-up data from the 68 colorectal cancer patients were analyzed and summarized. Time from surgery to patient mortality was recorded as the survival time. Kaplan-Meier survival curves were used to calculate 3-and 5-year survival rates, and univariate analysis was used to analyze the independent prognostic ability of PTEN, STAT3 and VEGF-C.

### Statistical analysis

SPSS 17.0 was used for statistical analysis. Numerical data were analyzed by the χ^2^ test and pairwise comparisons of multiple samples were analyzed by the segmentation method. One-way ANOVA was used to analyze the measurement data. Spearman’s rank correlation was used to analyze the correlation between PTEN, STAT3 and VEGF-C expression. The log-rank test was used to determine significant differences between survival rates. P-values of <0.05 were considered indicative of statistical significance.

## Results

### Relationship between PTEN, STAT3 and VEGF-C expression and clinicopathological features of colorectal carcinoma

**Figure 1:**
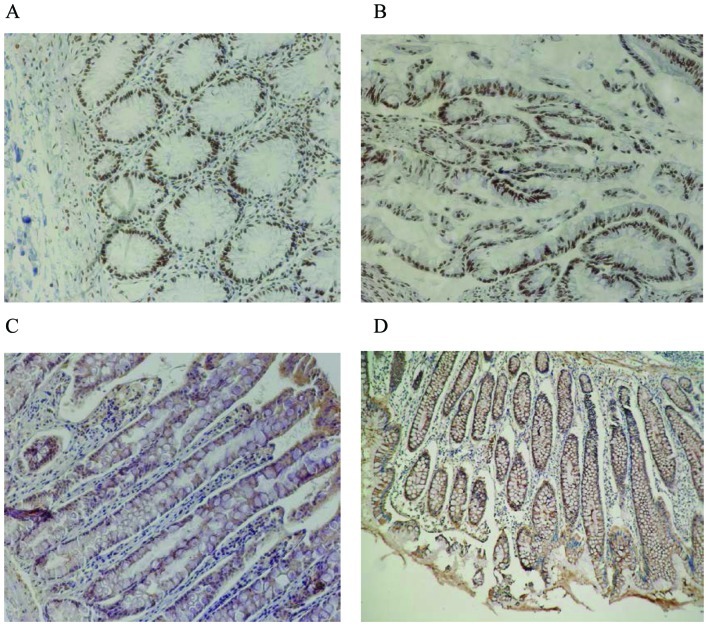
(A) PTEN immunohistochemistry in normal colorectal tissue. (B) PTEN immunohistochemistry in colorectal adenocarcinoma. (C) STAT3 immunohistochemistry in colorectal adenocarcinomas. (D) VEGF-C immunohistochemistry in colorectal adenocarcinoma. Hematoxylin counterstained; magnification, ×200.

PTEN protein expression was located in the nucleus, and STAT3 and VEGF-C protein expression was located in the cytoplasm, as yellow or brown granules (Fig. 1). The expression rates of PTEN, STAT3 and VEGF-C were 32.4, 60.3 and 63.2% in colorectal cancer samples, and 90.0, 0 and 0% in normal tissues, respectively; these differences were significant (p<0.05). Expression of PTEN and STAT3 protein was significantly related to the pathological grade of colorectal cancer (p=0.011 and 0.001, respectively) with no relationship between PTEN and STAT3 expression and tumor size, lymph node metastasis or clinical (TNM) stage. Expression of VEGF-C protein was significantly related to lymph node metastasis in colorectal cancer (p=0.002); with no relationship between VEGF-C and tumor size, pathological grade or clinical stage (Table I).

### Correlation between PTEN, STAT3 and VEGF-C expression in colorectal carcinoma

In colorectal carcinoma, PTEN and STAT3 protein expression was significantly negatively correlated (r=−0.402, p=0.001) as was PTEN and VEGF-C protein expression (r=−0.320, p=0.008). STAT3 and VEGF-C protein expression was positively correlated (r=0.254, p=0.036).

### Relationship of PTEN protein expression with colorectal cancer prognosis

**Figure 2:**
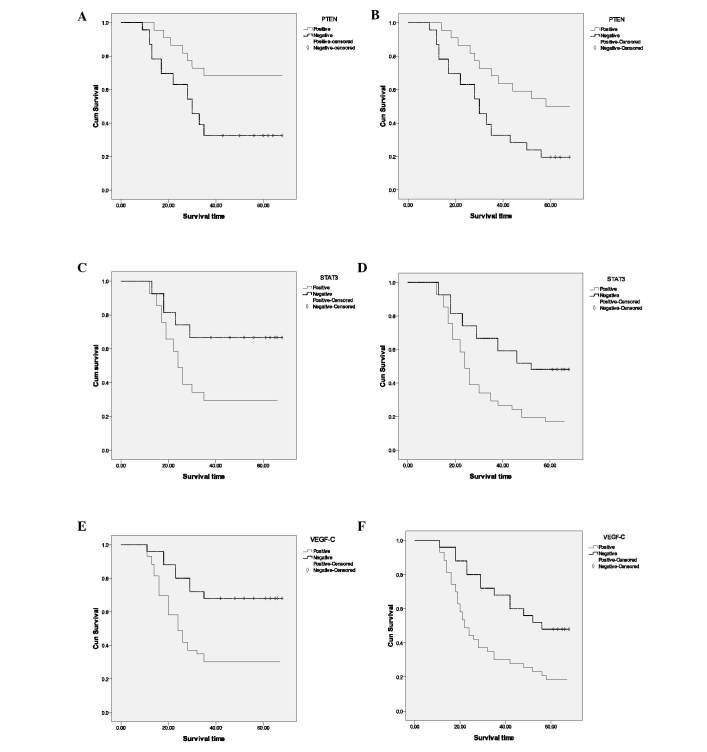
(A) The 3-year and (B) 5-year Kaplan-Meier survival curves for 68 patients with negative (n=46) and positive (n=22) PTEN expression. (C) The 3-year and (D) 5-year Kaplan-Meier survival curves for 68 patients with negative (n=27) and positive (n = 41) STAT3 expression. (E) The 3-year and (F) 5-year Kaplan-Meier survival curves for 68 patients with negative (n=25) and positive (n=43) VEGF-C expression.

The number of deaths and survival rate in PTEN protein-positive and -negative patients at 3 and 5 years are indicated in Table II. Median survival times for PTEN protein positive and negative patients were 38 and 23 months, respectively. Kaplan-Meier single factor survival analysis and the log-rank test indicated that the 3- and 5-year survival rates of PTEN protein-positive colorectal cancer patients were significantly higher than PTEN protein-negative patients (p=0.008, Fig. 2A and B).

### Relationship of STAT3 protein expression with colorectal cancer prognosis

The number of deaths and survival in STAT3 protein-positive and -negative patients at 3 and 5 years are indicated in Table II. Median survival times for STAT3 protein-positive and -negative patients were 21 and 29 months, respectively. Kaplan-Meier single factor survival analysis and the log-rank test indicated that the 3- and 5-year survival rates of STAT3 protein-positive colorectal cancer patients were significantly lower than STAT3 protein-negative patients (p=0.005, Fig. 2C and D).

### Relationship of VEGF-C protein expression with colorectal cancer prognosis

The number of deaths and survival in VEGF-C protein-positive and -negative patients at 3 and 5 years are shown in Table II. The median survival times for VEGF-C protein-positive and -negative patients were 20 and 28 months, respectively. Kaplan-Meier single factor survival analysis and the log-rank test indicated that the 3- and 5-year survival rates of VEGF-C protein-positive colorectal cancer patients were significantly lower than those of VEGF-C protein-negative patients (p=0.003, p=0.004, respectively; Fig. 2E and F).

### Prognostic factors in colorectal cancer

The 3- and 5-year survival rates and related prognostic factors were analyzed using Kaplan-Meier univariate survival analysis and the log-rank test. Pathological grade, clinical stage, and the presence of visible residual tumor and completion of regular chemotherapy after surgery were prognostic factors in colorectal carcinoma (Table III). To further determine the effects of independent prognostic factors in colorectal cancer, pathological grade, clinical stage, PTEN, STAT3 and VEGF-C protein expression, post-operative chemotherapy and residual tumor after surgery were analyzed using multivariate survival analysis. Clinical stage, postoperative chemotherapy, VEGF-C protein expression and histological grade were independent prognostic factors in colorectal cancer, while presence of residual tumor after surgery and PTEN and STAT3 protein expression were not independent prognostic factors (Table IV).

## Discussion

Numerous studies have investigated the prognostic factors in colorectal cancer; however, many studies have concentrated on a limited number of clinical stages, pathological grades or traditional prognostic factors, and have failed to correlate clinical stage with prognosis or make accurate prognostic judgments. Identification of new molecular biological markers to predict metastasis and recurrence is of major interest in cancer research. This study was designed to identify the correlation between PTEN, STAT3 and VEGF-C expression and prognostic ability in colorectal cancer.

The results of our study indicated that PTEN protein is expressed in colorectal cancer at significantly lower levels than in normal tissues and is indicative of poor prognosis, consistent with previous reports (7–9). The 3- and 5-year survival rates of patients with PTEN protein-positive colorectal cancer were higher than those of PTEN protein-negative patients. PTEN is a tumor-suppressor gene with lipid phosphatase activity, which negatively regulates the PI3K/AKT signaling pathway. Activation of PI3K/AKT signaling is linked to cell immortalization, promotes angiogenesis, and is involved in cell growth and differentiation (10). Hameed *et al* demonstrated that PTEN significantly inhibits the growth of esophageal cancer cells *in vitro* by inducing expression of the pro-apoptotic gene Bcl-2, and PTEN inhibits tumor growth *in vivo* (11). Absence of PTEN in colorectal cancer may affect the cell cycle and signal transduction pathways, thus promoting cell proliferation and tumorigenesis and leading to poorer prognosis. Clinical detection of PTEN protein expression may help to accurately predict prognosis in colorectal cancer and guide reasonable and effective clinical treatment.

Our findings demonstrate that STAT3 protein expression is significantly higher in colorectal cancer than in normal tissue and is associated with tumor grade. The 3- and 5-year survival rates of STAT3 protein-positive colorectal cancer patients were lower than the rates in STAT3 protein-negative patients, indicating that STAT3 may play a role in tumorigenesis, is related to the degree of malignancy, and influences the prognosis of colorectal cancer. STAT3 is an important member of the STAT family. Normally, activation of STAT3 signaling is subject to strict regulation, and STAT3 activation is related to malignant transformation (12). Zhang *et al* found that inhibitors of the JAK-2/STAT3 signaling pathway inhibit glioma proliferation, thereby preventing metastasis and invasion (13). STAT3 is abnormally activated and is related to tumor stage and prognosis in gastrointestinal tumors (14–16). STAT3 activation is linked to tumor progression, mainly through promotion of angiogenesis, anti-apoptotic effects and immune escape, and poor prognosis (17,18). Therefore, the detection of STAT3 expression in colorectal tumors may be a useful predictor of the degree of malignancy and prognosis, and may provide a novel target for cancer treatment.

VEGF-C was the first lymphatic growth stimulating factor to be reported. It regulates proliferation and differentiation of lymphatic endothelial cells and promotes tumor lymph node metastasis (19). In esophageal cancer, expression of VEGF-C is related to poor prognosis, and was suggested as an effective indicator for prediction of lymph node metastasis (20). In many tumors expression of VEGF-C is closely related to lymphatic vessel invasion, sentinel lymph node metastasis and distant metastasis and is indicative of poor prognosis (21). In colorectal cancer, we observed that VEGF-C expression was significantly increased compared to normal tissues and was related to lymph node metastasis.

The 3- and 5-year survival rates of VEGF-C protein-positive colorectal cancer patients were significantly lower than those of VEGF-C protein-negative patients, indicating VEGF-C has prognostic value in colorectal cancer. VEGF-C was also an independent prognostic factor of lymphatic system metastasis in bladder cancer and gastric cancer (22,23), and multivariate analysis in our study indicated that VEGF-C was also an independent prognostic factor in colorectal cancer. VEGF-C induces intracellular signal transduction to stimulate lymphatic endothelial cell proliferation via VEGF-R3 (19). VEGF-C may also directly activate VEGF-R3 in tumor cells to promote tumor growth and increase tumor invasiveness (24,25). By increasing formation of lymphatic vessels, VEGF-C increases the tumor cell contact with lymphatic vessels to promote metastasis. Additionally, VEGF-C increases lymphatic vessel permeability and tumor interstitial pressure, enhancing the risk of tumor cells entering lymphatic vessels and veins (26). Saharinen *et al* demonstrated that VEGF-C alters lymphatic endothelial cell adhesion properties, increases secretion of chemokines and cytokines and promotes tumor cell proliferation (27). Further investigation is required to determine the potential of VEGF-C as a potential target to reduce lymph node metastases and improve prognosis in colorectal cancer.

Numerous studies have shown that expression of PTEN, STAT3 and VEGF-C, which can regulate tumor cell growth, is closely related during tumor development. PTEN inhibits HIF-lα transcription factor activity, via PI3K/Akt/mTOR, reducing VEGF expression (28). PTEN and VEGF-C expression was negatively correlated in colorectal cancer, similar to the observations of Klatte *et al* in gastric cancer (4). VEGF is also a target gene of STAT3 (29), indicating that PTEN and STAT3 have a common target. In our study STAT3 and VEGF-C expression was also positively correlated. Evidence from the literature and our findings indicates that the expression of PTEN STAT3 and VEGF-C may be interdependent, and that each has their own role and prognostic value in colorectal cancer; downregulation of PTEN tumor suppressor activity and increased STAT3 and VEGF-C expression promote tumor growth, leading to increased invasion and metastasis in colorectal cancer.

In conclusion, PTEN, STAT3 and VEGF-C are prognostic factors in colorectal cancer and VEGF-C can be used as an independent prognostic factor. Combined detection of PTEN, STAT3 and VEGF-C expression may provide an index with which to determine the degree of malignancy, metastasis and prognosis in colorectal cancer and guide clinical treatment. Future research should focus on the methods by which to alter expression of PTEN, STAT3 and VEGF-C in order to provide new targets for clinical treatment, and to improve survival and quality of life of colorectal cancer patients.

## Figures and Tables

**Table I:** Relationship between clinicopathological features and expression of PTEN, STAT3 and VEGF-C in colorectal carcinoma.

		PTEN expression		STAT3 expression		VEGF-C expression	
Characteristics	n	n, rate (%)	P-value	n, rate (%)	P-value	n, rate (%)	P-value
Tumor size (cm)							
≤4	30	10 (33.3)	0.878	17 (56.7)	0.587	18 (60.0)	0.632
>4	38	12 (40.0)		24 (63.1)		25 (65.8)	
Grade							
Middle/low	48	20 (41.6)	0.011	23 (47.9)	0.001	29 (60.4)	0.662
High	20	2 (10.0)		18 (90.0)		14 (70.0)	
Node metastasis							
Positive	38	10 (26.3)	0.231	26 (68.4)	0.123	30 (78.9)	0.002
Negative	30	12 (40.0)		15 (50.0)		13 (43.3)	
Clinical stage							
I–II	32	12 (37.5)	0.392	18 (56.3)	0.520	19 (59.4)	0.534
III–IV	36	10 (27.8)		23 (63.8)		24 (66.7)	

**Table II:** Relationship of PTEN, STAT3 and VEGF-C protein expression with colorectal cancer prognosis.

		3-year			5-year			
Index	n	Mortality	Survival	%	Mortality	Survival	%	Median (months)
PTEN								
Positive	22	7	15	68.1	11	11	50.0	38
Negative	46	31	15	32.6	37	9	19.6	23
STAT3								
Positive	41	29	12	29.3	34	7	17.1	21
Negative	27	9	18	66.7	14	13	48.1	29
VEGF-C								
Positive	43	30	13	30.2	35	8	18.6	20
Negative	25	8	17	68.0	13	12	48.0	28

**Table III:** Survival rates of 68 patients with colorectal cancer stratified according to various factors.

Stratification	n	3-year (%)	5-year (%)
Grade			
High	20	30.0	10.0
Middle/low	48	50.0	37.5
Clinical stage			
I	13	100	76.9
II	19	68.4	52.6
III	24	45.8	29.1
IV	12	16.7	8.3
Residual tumor			
0 cm	26	73.0	65.4
<2 cm	30	40.0	16.7
≥2 cm	12	19.0	9.8
Chemotherapy			
No	12	16.7	8.3
Regular	39	56.4	48.7
Irregular	17	35.3	11.8

**Table IV:** Independent risk coefficients of independent prognostic factors in colorectal carcinoma determined using multivariate survival analysis.

Factor	Coefficient
Stage	2.128
Grade	1.765
Postoperative chemotherapy	0.610
VEGF-C expression	0.512
